# Cancer and Its Treatment

**Published:** 1892-06

**Authors:** 


Cancer and its Treatment. By Daniel Lewis, A. M., M. D., Ph. D., Surgeon to the New York Skin and Cancer Hospital; Professor of Surgery (Cancerous Diseases) in the New York Post-Graduate Medical School. The Physicians' Leisure Library. Price, cloth, 50 cents; paper, 25 cents. Detroit, Mich.: Geo. S. Davis. 1892.

The author of this book has had a large experience in the treatment of the disease in question, and we may, therefore, expect out of his hands a most interesting and useful book. The practical criticism to offer in regard to this treatise is that many of the subdivisions are too much condensed to be entirely satisfactory. Nevertheless, this could not well be otherwise in a book which must come within the limits required for a part of the Physicians' Leisure Library, and so this is not altogether the fault of the author. We hope that he will find occasion to elaborate the subject into a more complete treatise in the near future; meanwhile we commend this little volume to the careful examination of the profession. A few illustrations, two in color, add much to its interest.

